# Extra-Limites

**Published:** 1844-04-01

**Authors:** 


### EXTRA-LIMITES.

### INDIAN MEDICAL SERVICE.

*(To the Editor af the Medico-Chirurgical Review.)*

" If reward or emolument be the portion of a man borne so many years on the muster-roll, rather than of a character distinguished by activity, diligence, and discernment, the purpose of bounty is mistaken."—*Robert Jackson.*

Sir,—Since the days of the great American sea-serpent, which, like many of the American speeches, writings, and repudiation " new ways to pay old debts," had neither length nor breadth, substance nor shadow; nothing so voluminous yet volatile, so bulky yet impalpable, has appeared on this side the Atlantic and Indian Ocean, as your huge " Littlego"—of a letter from Bombay, dated " India, 20th August, 1843."

To meddle with this nobody of octocental humanity, this multitudinous nullity, this aggregate singularity, this polyandrian negation, would be like embracing a cloud, fencing with a water-spout, cudgelling with a hay-stack, or playing at singlestick with a feather-bed, aye, one stuffed with the down of Paddy-birds—an allusion which that writer will probably comprehend in the principle that birds of a feather, &c. There is no such a thing as impinging on such a nondescript. Strike as you may, all is hollow and defunct. There is no saliency, no elasticity of vitality. It has no sensible qualities, no voice, no smell. It is, and no mistake,—

" Sans teeth, sans eyes, sans taste, sans everything."

Strike high or low at it, there is no resiliency, no rebound. The inert mass, like the Indian Medical Boards, has neither body nor soul. It is without head or tail, or fact or argument, above the attention or invention of a camp-follower. Its horizon is limited to the nearest hedge, to the margin of Colaba Island, the surf at Madras, or the ditch of Fort William. But this will not do in the 19th Century. A mere gastric-juice, or rather bread-and-butter argument, is utterly unworthy of a professional and scientific body of men. We do not dislike bread-and-butter in their proper place, but we detest the smell of them when it flavours *esprit de corps.* It is indeed distressing to see a great flatulent fellow looming upon you as if 800 men were rolled into one, and able to articulate no other war-cry but *bread-and-butter!* Again, I say, this will not do in the 19th century. It has " an ancient and fish-like smell," that smacks of the olden time, when a voyage to India and back again took a twelvemonth. I would, therefore, recommend to your uneasy correspondent, to inform himself as to the working of MEDICAL Boards in India; and, secondly, to inform himself as to the best mode of administering the Medical Department of Armies. Had he thus qualified himself for the discussion of this matter, he would not have fallen into the preposterous platitude of assertion, that " they (the Boards) have never been convicted of inefficiency." To prove a negative is never a very agreeable operation. Obstructiveness, small cavilling jealousy, and dog-in-the-manger aptitude to snarl without the power to bite, these be the jewels that shine in the Ethiop's ear of Medical Boards.

Let me be believed, that I would not pass over argument had I found it. I have met not a scintilla of it in the letter I am noticing. *Seniores priores* is its principle. Fat things for dull age, and nothing for young merit, is its rule.

Optat Ephippia bis piger.

But heaven save the mark—live the Medical Board for ever—since, oh!

mighty discovery—"Boards have been found *most efficient* (!!!) in the management of their affairs; for there is a Government Council Board, a Revenue Board, a Military Board, a Clothing Board, &c." This is a clincher in Fluellen's style. "Look you—there is a river in Macedon, and a river in Monmouth, and salmons in both." Alas! for this lob-sided analogy, which is, in fact, no analogy at all. The members of all the Boards he alludes to, save the medical, are selective. In not one of them is a member such by seniority, nor can any servant claim membership by right of seniority. The Government nominates members of Council, and members of the Board of Revenue, &c. Fancy a Supreme Council of the State, where not merit, not high character, not generous zeal, not high mindedness, not comprehensive ability, and talent, and reputation, but *seniority* alone gave the claim to nomination! Does not the very idea sound absurd and inconsequential? It is not a bit more absurd and inconsequential than the seniority principle alone as the controlling power in a body of eight hundred Army Surgeons.

Finally, let me advise your Correspondent never to attribute motives, more especially when his natural bias blunders only on bad ones. Let him meet the facts of officers and gentlemen with facts. *Au reste,* I would not do such a discredit to Mr. Annesley and Mr. Martin as to attempt a defence of their honored names against such a Triton of the minnows as this $\frac{1}{800}$th fraction of humanity. When a bit of steel could speak, it said to the pugnacious reptile as it crept away with bleeding tongue—"Viper thou gnawest at a file."

ANOTHER OF THE EIGHT HUNDRED.

---

